# An objective measure of hyperactivity aspects with compressed webcam video

**DOI:** 10.1186/s13034-015-0076-1

**Published:** 2015-09-10

**Authors:** Thomas Wehrmann, Jörg Michael Müller

**Affiliations:** Department of Child and Adolescent Psychiatry, University Hospital Münster, Schmeddingstrasse 50, 48149 Münster, Germany

**Keywords:** ADHD, Hyperactivity, Objective measure, Physical activity, Video

## Abstract

**Background:**

Objective measures of physical activity are currently not considered in clinical guidelines for the assessment of hyperactivity in the context of Attention-Deficit/Hyperactivity Disorder (ADHD) due to low and inconsistent associations between clinical ratings, missing age-related norm data and high technical requirements.

**Methods:**

This pilot study introduces a new objective measure for physical activity using compressed webcam video footage, which should be less affected by age-related variables. A pre-test established a preliminary standard procedure for testing a clinical sample of 39 children aged 6–16 years (21 with a clinical ADHD diagnosis, 18 without). Subjects were filmed for 6 min while solving a standardized cognitive performance task. Our webcam video-based *video*-*activity score* was compared with respect to two independent video-based movement ratings by students, ratings of *Inattentiveness*, *Hyperactivity* and *Impulsivity* by clinicians (DCL-ADHS) giving a clinical diagnosis of ADHD and parents (FBB-ADHD) and physical features (age, weight, height, BMI) using mean scores, correlations and multiple regression.

**Results:**

Our *video*-*activity score* showed a high agreement (r = 0.81) with video-based movement ratings, but also considerable associations with age-related physical attributes. After controlling for age-related confounders, the *video*-*activity score* showed not the expected association with clinicians’ or parents’ hyperactivity ratings.

**Conclusions:**

Our preliminary conclusion is that our *video*-*activity score* assesses physical activity but not specific information related to hyperactivity. The general problem of defining and assessing hyperactivity with objective criteria remains.

**Electronic supplementary material:**

The online version of this article (doi:10.1186/s13034-015-0076-1) contains supplementary material, which is available to authorized users.

## Background

This paper introduces a new objective measure for hyperactivity using compressed webcam-video footage. The method is introduced and explored for the assessment of hyperactivity, and it may contribute objective information for the assessment of *Attention-deficit/hyperactivity disorder* (ADHD).

*Attention deficit hyperactivity disorder, hyperactivity* ADHD is the most common neurobehavioral disorder among children, and the reported prevalence rates vary from 2 to 18 %, depending on several factors, e.g., the selected classification system and the studied population [1]. The prevalence reported in a newer European study that was based on parent and teacher reports was 5.2 % [2]. The American Psychological Association characterizes ADHD in the DSM-5 as a persistent pattern of inattention and/or hyperactivity-impulsivity that interferes with function or development [3, 4]. In the following study, we discuss several approaches for assessing ADHD symptoms; we focus on hyperactivity, which represents the main behavioral criteria in this paper.

Clinical guidelines suggest a clinical evaluation by experienced clinicians, which could comprise personal observations, a clinical interview, and self- and parental reports by questionnaires for the assessment of ADHD and hyperactivity [5, 6]. Notably, physical or neurobiological markers of hyperactivity are actually not suggested due to a low agreement between physical or neurobiological markers and clinical observation, which has been frequently reported. In fact, all respected situational facets during clinical evaluation include a subjective judgment by the clinician. This seems to be one source for the reported disagreement across raters not only across clinicians but also across all different rater-types, such as parents, teachers, or blinded raters [7, 8]. A second source of disagreement has its origin in the strong dependency between age and physical activity that is already observable within one cohort of the same age. For example, children who are relatively old for their school grade have lower and children who are relatively young for their school grade have a higher incidence of ADHD [9].

In the following we present a brief overview to highlight the pros and cons of different assessment approaches, with a focus on hyperactivity. This should facilitate an understanding of the small overlap across the different methods and underline the advantages related to our approach. However, it is important to note that, as yet, there is neither an accepted gold standard nor are there any main criteria capable of comparing validity coefficients.

*Rating scales for ADHD* There are a variety of rating scales to assess ADHD symptoms and hyperactivity using ICD-10 or DSM-5 criteria, e.g., the *Conners Rating Scale* (CRS), the *Vanderbilt Rating Scale*, and the *ADHD*-*Self Report System*. Most of these rating scales capture hyperactivity as a core symptom of ADHD [10, 11]. Rating scales have the advantage of a high face-validity because the DSM-5 proclaimed contents are often explicitly named within the item formulation. The standardized questions allow for an amplification of the information basis by using multiple informants, which contributes to an assessment of hyperactivity in a standardized way [10]. Rating scales have further advantages, such as cost effectiveness, the fact that they can be administered by mail or in an online assessment, or the possibility of being discussed with clinicians. The main disadvantages are low inter-rater agreements [8]. For instance, Wolraich et al. found poor inter-rater agreement for the Vanderbilt Attention Deficit Hyperactivity Disorder Rating Scale, a Questionnaire also including DSM-IV criteria (9 items for inattention and 9 for hyperactivity/impulsivity), in a 243 case sample. Correlations in syndrome counts between parent and teacher ratings ranged from only r = 0.27 for hyperactivity/impulsivity to r = 0.34 for inattention. Breuer et al. found a correlation between two teacher ratings of r = 0.65 for hyperactivity/impulsivity and r = 0.74 for inattention, with a sample of 50 children aged 6–16 when both ratings depicted the same situation. The correlations between parent and teacher ratings were r = 0.42 for hyperactivity/impulsivity and r = 0.43 for Inattention; the sample consisted of 78 children aged 6–16 [7]. However, these described associations have not been controlled for age.

### Capturing physical activity

In addition to the clinical meaning of hyperactivity, we distinguish physical activity as an inevitable behavioral correlate. Here, we use the term “Physical Activity” in a generic way, depicting every physical movement produced by muscle activity that increases the metabolic rate at rest [12]. Pure physical activity can be registered in many ways, for example, by heart frequency, burnt calories or metabolic equivalents, which compare the increase of the metabolic rest rate [13]. Physical activity consists of a nearly infinite variety of single movements. Each of the following methods emphasizes a different subsample of the manifold possibilities measuring the behavioral correlates of hyperactivity.

*Accelerometers* Accelerometers, in general, quantify changes (frequency and magnitude) in the moving direction of a single selected body location in two- or three-dimensions. Accelerometers consist of a small recording unit, which is attached to the wrist or the hip, making it flexible and applicable across many settings and conditions [14, 15]. However, accelerometer data need a considerable amount of time for the assessment of activity, ranging from 2 h [16] up to data collection over 6 days [17]. After data collection, considerable effort is needed for Integration and filtering to avoid bias from motion from unintended sources to achieve reliability coefficients, which range from r = 0.81 to r = 0.84. Activity ‘scoring’ has been suggested, e.g., by “G units” [16] or “activity counts”, which can be compared to metabolic equivalents (MET) depending upon the research question. MET relate metabolism rates to bodyweight and are developed to compare different levels of physical activity while disentangling the strong relationship between age, physical load, subjects’ occupations and physical measures of activity. Ignoring such basic relationships could lead to artificial differences in group activity levels [18].

The flexibility in setting, application and scoring of activity quantity leads to a problem of developing normative data and achieving comparability. Accelerometers have therefore been applied only in research studies (evaluation of drug effects [19] or in analyses of situational factors on the activity level [20]), but not in clinical assessments. In their review of accelerometers, including 32 studies, De Vries et al. (2009) described that only two motion sensors (Actigraph and Caltrac) have been examined for reliability and validity in different age groups (2–18 year) but not across different age groups [15]. However, differences between children with ADHD and controls [21, 22] were detected solely for age-homogenous groups of six-year-old children for an assessment of up to 24 h [23]. Probably the most important disadvantage of accelerometers is a low to missing validity to rating scales or clinical evaluations. Dabkowska et al. (2007) found no evidentiary correlation between parent ratings for ADHD and *Actigraph* data in a sample of 21 children who wore an actigraph for 3 days [24], and Dane et al. (2000) published correlations between *Actigraph* data and expert ratings ranging from r = −0.24 to r = 0.09 [25].

*Infrared motion tracking* The infrared motion tracking (IMT) system is based on a video recording of an infrared strobe camera that records the two dimensional movement of reflective patches attached to subjects’ head and shoulders. This technique uses four (instead of one, see accelerometers) standardized locations for the detection of movement. Additionally the assessment takes place in a highly artificial standardized setting (Teicher et al. [26]) during a continuous performance visual task (CPT). Each CPT session took 5 min and was repeated three times within 30 min. The derived movement scores detected significant differences during a CPT between 18 boys with ADHD and 11 without ADHD. Children with ADHD moved their heads 2.3 times more often in a 3.8-fold greater area. The main captured parameters from IMT were position changes and the complexity of movement [27, 28]. Similar to the accelerator measures, the IMT showed no significant correlations between head movement and parent ratings in the overactivity/inattention of the IOWA-Conners Scale or parent ratings in the overactivity of the abbreviated Conners Scale [26]. Note that the IMT has been applied in only a few studies.

### Aims of the study

This article aims to introduce a simple, reliable and valid method to assess hyperactivity objectively by using webcam footage and video compression. We assume that physical activity—recorded by webcam videos—impacts the footage file size after compression. We expect high agreement (>0.60) between our file size score and independent movement ratings based on the same video footage. Furthermore, we expect significant and substantial agreement (>0.30) with the hyperactivity scale scores of clinical ratings by standardized questionnaires and, hopefully, to parental ratings.

## Methods

*A new video*-*based objective approach to assess physical activity* Our measure for physical activity is based on the idea that compression techniques in general try to reduce the amount of storage by eliminating unnecessary information [29]. In the case of video compression, a sequence of frozen objects contains the minimal amount of information because every subsequent picture (or frame) looks like the initial one. In this case—for example—the footage contains thirty frames per second before, and only one (the initial frame) after, compression. All of the following frames are deleted because they do not contain additional information. This reduces the file size. The more changes between single frames there are, the fewer frames can be deleted. This leads to an increase in file size. In our approach, physical activity is represented by the movement or stationary position of our subject. Rest causes small file sizes (minimum of additional information), and movement causes an increase in the file size, as stated above. The necessary setting prerequisites are a fixed webcam with an unmoving background and a moving object. The file size per minute can therefore serve as an objective, quantified measure regarding physical activity and has been applied in a different context for the assessment of physical activity in non-human primates by Togasaki et al. [30].

*Preparation of experiments* In our first experiment (henceforth termed the *Pre*-*Test*), we tested our basic hypothesized relationship between simulated moving objects and the file size, and we checked for several technical conditions (e.g., different webcam products, figure/ground texture, compression techniques and so on) to detect confounders having an unintended impact on the file size in our video capture. The *Pre*-*Test*, therefore, yielded the first set of standardizations, which can be used in the subsequent clinical experiment.

### Pre-Test

*Target* The first author created five sequences as examples for an objective movement pattern, containing different settings. All five sequences were created with 30 frames per second using Adobe™ Flash CS3 Professional, with a resolution of 1024 × 860 pixels. We simulated the following conditions: (1) no movement (white background without any moving object as a baseline for white noise); (2) movement of a black circle on a white background; (3) like condition (2), but the texture in the moving object simulates the influence of different clothing textures; (4) like condition (2), but with texture in the background to simulate different room conditions; and (5) like condition (4), but with texture in the moving object. Conditions (2) to (5) used the same movement pattern.

*Webcam* We examined several webcams and selected the Microsoft™ (LifeCam VX-3000, v1.0) webcam because of its superior discrimination rates (not reported here in detail because of space limitations). The footage was captured using the onboard software for the aforementioned camera and the highest recording quality and solution possible to manipulate, in a subsequent second step, the best resolution for discrimination.

*Setting* The camera was installed on a table in front of a 50 Hz LCD-Monitor and adjusted to the screen. The created sample sequences were shown on the screen and captured by our camera.

*Video compression* We cut and compressed each video using X-Media-Recode, an Open Source tool for video compression [31]. The output format was 3gp, a container format for mobile surfaces, using the MPEG-4 codec [32]. Captured films were cut into pieces of 6, 12, 18, 24, 30, 36, 42, 48, 54 and 60 s. This procedure was executed twice, with two differing starting points.

*File size measure of activity* Each pixel of the web cam sensor worked as its own movement sensor. In our Pre-Test, we determined a resolution of 176 × 144 pixels. Therefore, we obtained 25.344 movement sensors instead of four (in case of IMT) or less (*Actigraphy*). In practice, approximately one-fifth of all sensors assessed our test object, the others assessed the background. After a full recording of a movement condition, approximately 80 % of all pixel sensors were used to assess changes or activity because the object moved through different areas. Each full-length video was cut (6, 12, 18,… 60 s) and compressed with a 176 × 144 pixel-resolution and 30 frames per second (fps). The data were handled on a Mac Book with a 2.26 GHz Intel™ Core 2 Duo processor, 4 GB DDR3-RAM, a NVIDIA™ GeForce 9400 M, and Windows™ XP. We assessed the file size given in the Windows XP explorer because the Apple OS reported only rounded estimations of the real file size.

**Fig. 1:**
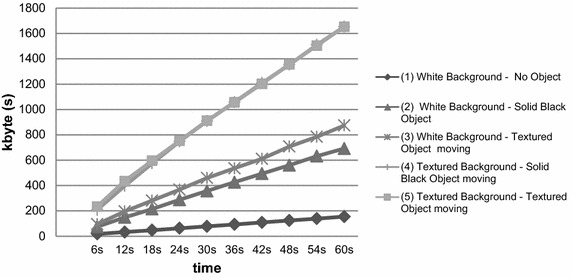
File size, in kbyte, as a function of time for the five conditions (see legend)

*Results of the Pre*-*Test* Figure 1 shows the mean file sizes for each condition and repeated sequences as a function of time and our five conditions.

*Discussion of the Pre*-*Test experiment* The first step was to check our assumption that additional movement directly increases the file size and determines which conditions would provide the best activity score. Figure 1 shows an acceptably low level of noise influences in capturing a white background (condition 1), which is a basic proof of the general idea of an increased file size caused by a moving object (condition 2 compared to 1), the influence of texture of the moving circle (condition 3 vs 2 and 5 vs 4) and the influence of the texture of the background (condition 4 vs 2 and 5 vs 3). The results of our *Pre*-*Test* support the development of a preliminary procedure to compute an *activity score* (see below).

### Clinical experiment

*Procedure* We recruited our sample from patients of the Department of Child and Adolescent Psychiatry at the University Hospital of Muenster and from a settled Child Psychiatrist in Muenster over a period of 6 months (October 2010 to March 2011). Each child in our sample was seen and diagnosed by a child psychiatrist. The criteria for exclusion were medication use, mental disability, reduced intelligence (IQ <80), schizophrenia and suicidal tendencies. Based on the diagnoses, our clinical control sample was without hyperactivity and featured the following diagnoses (the frequencies are presented in the parentheses): Predominantly compulsive acts [obsessional rituals] (1), Adjustment disorders (2), Persistent somatoform pain disorder (2), Anorexia nervosa (3), Acute and transient psychotic disorder, unspecified (1), Other habit and impulse disorders (1), Sibling rivalry disorder (1), and Other childhood emotional disorders (6). The sample of clinical disorders was enriched by a sample with Hyperactivity such that the final sample should show a sufficient variation in hyperactivity for our dimensional validity approach (see below). The Hyperactivity sample exhibited the following diagnoses: Disturbance of activity and attention (12), Hyperkinetic conduct disorder (7), and Hyperkinetic disorder, unspecified (3). After obtaining informed consent, the testing took place in two rooms. For each child, the therapist filled out the DCL-ADHS [33] independently of the testing of the child. The accompanying parent completed a sociodemographic questionnaire and a FBB-ADHS questionnaire (see below). This study was approved by the Ethics Committee of the University of Muenster .

*Sociodemographic description* Our sample consisted of 39 children (12 girls and 27 boys) with an age range from 6 to 16 years. The mean ages not only for the total sample but also for the ADHD and clinical control subsamples are presented in Additional file 1: Table 1. Thirty-eight children (97.4 %) were German, and one (2.6 %) was a non-EU national. A total of 69.2 % of the children (N = 27) lived with both parents, and 30.8 % (N = 12) lived in a single-parent family. Only 3 children (7.7 %) were the sole child in their family, 23 children (59.0 %) had one sibling, and 13 (33.3 %) had two or more siblings. Six children (15.4 %) were in grammar school (12–13 years of education), 15 (38.4 %) in secondary modern school (9-10 years of education), 15 (38.5 %) in primary school (4 years of education) and 3 visited a school for handicapped children. The sample showed the expected variation in physical attributes for children between 6 and 16 years with respect to height, weight and body-mass-index (see Additional file 1: Table 1).

*Task for the participants* Hyperactivity in the context of ADHD has frequently been studied in experimental conditions that have focused on the processing visual stimuli, e.g., within the CPT (see above). However, such settings seem inappropriate for our research aims for several reasons. First, we sought to observe increased physical activity; thus, the subject needed many options for showing such increased activity. Unfortunately, many experimental settings seek to prevent physical activity because the investigators view it as source of error, for example, while observing neuronal responses. Second, we aimed to model a setting with greater context-specific validity. Our context is characterized by listening carefully to someone and is thus similar to, for example, listening to a teacher in a classroom [34] or listening to a caregiver; thus, we used auditory stimuli. Previous studies have observed performance deficits related to both auditory and visual stimuli [35–38] irrespective of the presumed ADHD-related deficits of impaired central executive or phonological storage/rehearsal processes. Third, we aimed to design a setting that involved repeated durations of waiting. The theory of optimal stimulation suggests that hyperactive children with high stimulation thresholds exhibit stimulation-seeking behaviors in situations with low amounts of stimulation. Stimulation-seeking behavior is characterized by increased physical activity [39, 40]. Additionally, we expected to observe increased hyperactivity behavior due to the delay aversion of children with ADHD particularly when that delay period cannot be altered [41]. Collectively, these findings suggest that hyperactivity can be observed in an auditory cognitive task that was created based on the standardized “repeating numbers” task from the Hamburg-Wechsler Intelligence Test-IV [42] and presented via taped audio. The subjects were instructed to remain seated on a chair without an armrest during the test. The audio playback began with an introduction that provided two examples (e.g., instructions: “Please repeat the following numbers: 1, 2” followed by a time that was sufficient for the subject to repeat both numbers). During the task, the participant has to wait and/or to listen most of the time to the playback to uncover fidgeting [27] or an increase in the level of general activity [43]. The audio instruction took 6 min and 56 s. This standardized task ensured a video record length of a minimum of 6 full minutes.

**Fig. 2:**
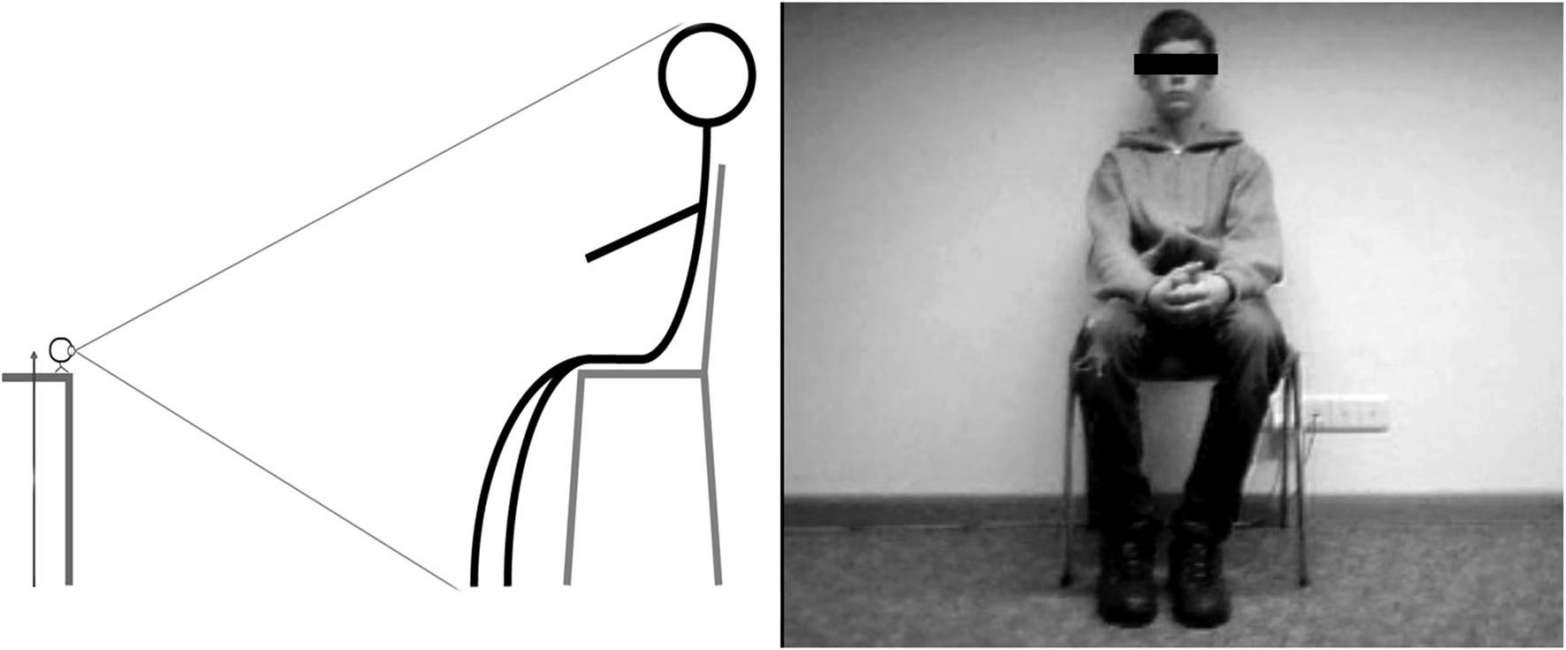
Webcam recording setting. Figure 1 shows the general setup used to record movement. The camera adjustment is shown on the *left*. The distance to each subject was detected by barely capturing the scalp and feet while placing the camera on a table approximately 50 cm above the ground. On the *right side*, a picture of a sequence is shown. The camera was adjusted so that each subject was sitting roughly in the middle of the frame

*Video recording setup* Figure 2 shows our general setup. The webcam was placed on a Table 50 cm above the ground and directly in front of the seated subjects to assure a frontal video capture of each subject. It was adjusted so that the feet and the scalp were barely in the picture, with the subject in the middle. This setting was used for two reasons: First, the differing body height in the sample should not influence the measure of change in this way and bigger subjects fill in the screen more than small children. Without these precautions, a small amount of movement from large subjects could lead to more changes in the file size compared to a larger amount of movement from small subjects. Thus, differences in height, weight and age should be reduced, and the measure should be comparable for different subsamples. Second, this standardization should lead to a fast and easy, but comparable, standard setup. The video background was a white wall, and testing was conducted in daylight conditions. The investigator hid behind the computer, without permitting eye contact and remained quiet to prevent additional influences during the test. The video capture was started simultaneously with the audio recording of the task to synchronize the video capture.

*Video*-*activity score* As mentioned above, the results of our *Pre*-*Test* enable us to compute a preliminary *activity score* as described next. The influence of the background texture was eliminated in the style of the technique of digital subtraction angiography [44]. This was achieved by recording the setting without a participant and subtracting the file size of this sequence from the clinical video file size (compare *Pre*-*Test* condition (1), “white noise”). The difference only represented the file size produced by the moving participant itself, without differences caused through white noise of different backgrounds. A second improvement was to reduce bias from flickering. Flickering means single pixels switch brightness or color and is reduced in monochromatic and plain areas. Therefore, the record setting contained a white wall. Moreover, we reduced the pixel amount in our compressed sequences to minimize flickering. The video records of each child were cut and compressed with X-Media-Recode into a six minute sequence. Each had a resolution of 128 × 96 pixels (=12.288 sensors to record activity). Additionally, all color and audio information were deleted. The file size was divided by the number of seconds to yield a time-independent *video*-*activity score.* Our *video*-*activity score* is based on the complete record, and two split-half *activity scores* were built in an odd–even version by summing the file sizes for the first, third and fifth minute to build an odd-*activity score*. The even-*activity score* summed the second, fourth and sixth minutes. The odd–even reliability of both was r = 0.97.

*Video*-*based movement ratings of captured activity* We expected interpretational problems in the case of a missing association between a clinical expert rating of hyperactivity and our *video*-*activity score*. The *video*-*activity score* may not assess ‘movement’ in the eye of human observers or, alternatively, may indicate a missing representativeness inside the testing situation to behavior outside the testing situation, which is assessed by questionnaires (see below). Therefore, we assessed ‘movement’ by two independent raters based on our webcam footage. All of the videos were cut into one minute sequences, ordered randomly, and were then rated by two students. Instructions were: “rate ‘the quantity of movement’ on a scale from 0 (=no movement) to 4 (=much movement) separated for the head, body, arms and legs.” These four detailed ratings were summed to a movement rating for each video minute and resulted in a total of n = 234 ratings (39 subject × 6 min) for each rater. We aggregated the ratings for the 6 min across one child to yield a ‘one-child movement score’ from each rater. The correlation between both rater scores was r = 0.97 (p < 0.001, N = 39). To simplify further statistics, we aggregated both ratings to one *movement rating*.

*Questionnaire measures for activity* The FBB-ADHS is a disorder-specific standardized and normed questionnaire from the DISYPS-II for children [37] based on a parent report. The FBB-ADHS assesses the components by separated scales of *Inattention* (9 items), *Hyperactivity* (7 items, alpha = 0.86) and *Impulsivity* (4 items) in the German language. In a large sample of 2863 children [11], the questionnaire showed satisfying reliability and convergent validity, e.g., with the Conners Rating Scale [45] or the *Strengths and Difficulties Questionnaire* (SDQ-hyperactivity; r = 0.69) [46]. Both, the FBB-ADHS and the CRS showed acceptable factorial validity in a confirmatory factor analysis and internal consistency, with Cronbach’s α ranging from 0.84 for CRS to 0.90 for FBB-ADHS (see [11] for further information). The DCL-ADHS is the expert version of the FBB-ADHS, except that two hyperactivity items are missing (“describes a feeling of internal arousal” and “is often activated or acts as driven”) [33]. An ICD-10 ADHD diagnosis is derived from the DCL-ADHS. The internal consistency for *Hyperactivity* is alpha = 0.91 [7].

*Validity approach* The *tenth International Classification of Diseases* (ICD-10) describes hyperactivity in terms of being *disorganized* and *ill*-*regulated*, but highlights quantitative aspects, such as being *excessive* [47], including *fidgeting*, *seat*-*leaving*, *being “on the go”* and *running around* or *talking excessively* in *improper* situations (see also the fifth *Diagnostics and Statistical Manual of Mental Disorders, DSM*-*5*), which impact normal living [3, 47]. As noted in the introduction, there is no gold standard for the assessment of ADHD, especially hyperactivity. In our understanding, hyperactivity is primarily a clinical term, but with a mandatory background of increased (hyper) physical activity. Given by *DSM*-*5* (“excessive motor activity when it is not appropriate”), a recognizable amount of increased activity has to be evaluated within a subjective interpretation. This interpretation takes into account situational specificity, familial context information and normal physical activity (see DSM-5 [3]) to yield a relative and integrative judgment about clinical relevance, severity and syndrome burden. We therefore consider the clinical diagnoses (also considering the standardized questionnaire with parental report plus own observations) just for descriptive purposes to examine and illustrate subsample differences in DCL-ADHD, FBB-ADHD and the *video*-*activity score.* Our validity approach is, in total, threefold.

In a first step, we validated the *video*-*activity score* by movement ratings to assure that it assessed ‘physical activity’. In a second step, we compared the mean scores of questionnaire-based hyperactivity ratings and control variables between the ADHD and control subsample based on a categorial diagnosis of experts. We additionally report the association between all activity-related measures and control variables. In the third and most important step, we examined whether the expert rating (DCL-ADHD; scale Hyperactivity), which was controlled for age and BMI within a multiple regression analysis, was associated with a high *video*-*activity score*. This is based on our assumption that the expert ratings assess no age-dependent activity, but focus on hyperactivity-specific movements. It is also desirable that the *video*-*activity score* is substantially associated with the parental *Hyperactivity* score from the FBB-ADHD to achieve a high face-validity for the parents. We hoped to observe only negligible associations with age (and related variables, such as weight and height) because the record setting aimed to reduce those influences by its adjustment to subject’s body height (see Fig. 2). Thus, all height-related factors, such as age or weight, should also be adjusted. The BMI is an already height-adjusted measure, and its visual importance for the *video*-*activity score* is unclear. Skinny children may show a higher *video*-*activity score* because of quicker movements (=more pixel changes), and this may add an incremental validity above the influence of age or height. However, children with a greater BMI may move slower, producing more pixel changes through their larger body surface. In the end, both effects may counterbalance each other, and we cannot predict a positive or negative association with our *video*-*activity score*.

*Statistical analysis for the clinical experiment* The first data examination reports the means and standard deviation of the *video*-*activity scores* together with the subscales *Inattentiveness*, *Hyperactivity* and *Impulsivity* based on clinical and parental ratings, along with the child physical attributes for the total sample and separately for the ADHD and control subsamples. We also report the Pearson correlation between our *video*-*activity score, movement rating,* questionnaire-based hyperactivity ratings from the clinical experts and parents and the physical attributes of the child. Finally, we analyze, within a multiple regression, the validity to our *video*-*activity score*. We selected only the most important variables because of the limited number of cases in this pilot-study. We included the age and *BMI*, clinical expert and parental rating scale of hyperactivity. We excluded the movement rating from the testing situation because we were interested in the validity of the *videoactivity score* outside of our testing situation. Note that one FBB-ADHS questionnaire and one DCL-ADHS expert checklist were missing, but not for the same child. Because this is a pilot study with a limited number of participants, we accepted a Type I error of p < 0.10 to identify a trend and p < 0.05 for significance.

## Results

### Descriptive statistics

The basic descriptive statistics for our *video*-*activity scores*, the video-based movement ratings, the clinical and parental ratings and the physical attributes are reported in Additional file 1: Table 1. To describe the observed variation in all hyperactivity measures and control variables, we present the mean score differences between the ADHD and the clinical control subsample along with independent t-tests and Cohens’ *d*, while focusing on the dimensional validity approach via multiple-regression analysis. Our *video*-*activity score* shows a considerable range between the minimum and maximum score, and the Kolmogorov–Smirnov test on normal distribution was not significant (df = 39, p = 0.138). As expected, both subsamples differed in their *video*-*activity score* and their movement ratings, with a greater effect size for the movement rating. Furthermore, the expert and parental ratings for the *Inattention*, *Hyperactivity* and *Impulsivity* constructs show subsample differences, but so did the physical control variables. Therefore, the observed mean score differences have to be interpreted with caution, and differences in the control variables have to be controlled by a multiple regression analysis. In general, we observed sufficient variation for subsequent bivariate and later multivariate analyses in all variables. Such analysis is preceded by the presentation of a correlation matrix to examine the descriptive strength of bivariate associations.

### Correlations between video-activity scores and other variables

We observe in Additional file 1: Table 2 an expectedly high positive correlation between the movement rating and our *video*-*activity score* (correlation inside the testing situation). However, we observe no substantial correlations between the *video*-*activity scores* and the clinical expert or parental *Hyperactivity* ratings. Furthermore, there was an unexpected moderate association of our *video*-*activity score* with age, height, weight and BMI.

Note that upon further analysis, the multiple regression is affected by the high intercorrelation between age, height, weight and BMI because of their multicollinearity. A similar problem is related to the correlation between the expert rated subscale *Hyperactivity* to *Inattention (r* = 0*.70, p* < 0*.01)* and *Impulsivity* (r = 0.80, p < 0.01) and also for the parental rating of *Hyperactivity* to *Inattention (r* = *0.67, p* < *0.01)* and *Impulsivity* (r = 0.69, p < 0.01). Such may increase the problem of parameter estimation. In general, the correlation in Additional file 1: Table 2 should be carefully interpreted because each association is not controlled for all of the other associations. We performed this by the following multiple regression analysis.

### Multiple regression analysis

The unexpectedly high dependency between the *video*-*activity score* and age-related variables underlines the need to control for age to examine the relationship between the *video*-*activity score* and the clinical expert and parental ratings. We assume that the clinical expert rating is already adjusted for age influences. The strong influence of age enables us to answer additional questions, such as how much “movement” variance is explained by age in a sample of 6–16-year-old children and how much is caused by hyperactivity (assessed by the DCL-Hyperactivity scale from experts). The knowledge of this proportion may help to estimate the necessary sample size of experiments to disentangle age and hyperactivity-related movement variance more accurately. The results of the multiple regression analysis and related regression coefficients are given in Additional file 1: Table 3. This model shows an R = 0.575, which explains the R^2^ = 33.0 % of the variance in file size (adjusted R^2^ = 24.7 %), which is significant with F(4,32) = 3,95, p = 0.010.

## Discussion

### Conceptual evaluation of the webcam assessment approach

This paper introduced and examined a new objective activity assessment procedure using the file sizes of compressed video captured from a standardized setting that should provoke hyperactivity behaviors. Our approach was developed with the experience of previously attempts to validate methods for the objective assessment of hyperactivity, e.g., *Actigraphy* and IMT. Generally, hyperactive behavior is not easily observable. Amongst other things, the assessment of hyperactive behavior requires a relatively long recording time. Teicher et al. [26] needed a three-time repetition of the CPT within 30 min, and some accelerometer studies achieved reliable results only after several days of recording. This assessment problem was resolved in our study by a standardized cognitive performance task in a comparatively short time, focusing on behaviors relevant to hyperactivity. A disadvantage of this procedure is the need to demonstrate external validity, which was resolved by expert and parental ratings from outside the testing situation.

A second validation problem of accelerometer based methods was their low agreement to clinical ratings. This was partly explained by the assumed higher influence of age-related activity compared to hyperactivity specific behavior. As already mentioned above, we tried to reduce or eliminate age-related physical activity in the best case by our recording setting (see Fig. 1). A third problem of accelerometer-derived scores is their unknown face validity with independent observers. The accelerometer technique does not permit a concurrent validation on the same material. This is probably a unique advantage of the video-compression method because it allows for the comparison of the *video*-*activity score* with independent movement ratings based on the same video material.

### Evaluation of the instruments

In general, the precondition to assess hyperactivity by reliable measures is given by the high variation and the very high split-half (odd–even) of r = 0.97 of the *video*-*activity score.* Furthermore, a similar high interrater agreement of r = 0.97 for the *movement rating* and reasonable scale intercorrelation between the established reliable, validated and normed questionnaires, DCL-ADHD and FBB-ADHD, used to assess *Hyperactivity, Inattention* and *Impulsivity* by clinical experts or parents was found. However, *Hyperactivity* rating by experts were, as expected, only moderately associated with the parental ratings.

### Validity of the video-activity score

In the *Pre*-*Test,* we successfully established a preliminary procedure to scale physical activity by the file size of compressed video footage. In the clinical experiment, we aimed to validate our new objective physical activity score. The mean scores in each instrument showed the expected direction for the ADHD and control subsamples. Additional file 1: Table 1 shows the increased scores from clinical experts from a standardized and reliable instrument. In addition, the parental ratings and movement ratings verified differences between the ADHD and control subsamples. These subsample differences are also observable for our *video*-*activity score*. We previously noted that the findings in Additional file 1: Table 1 should be interpreted with caution, as both subsamples were not controlled for physical differences. In Additional file 1: Table 2, we examined the bivariate relationship between the *video*-*activity score* and the validity indicators inside (*movement rating*) and outside (clinical expert and parental hyperactivity rating) the clinical experiment. We successfully demonstrated that our *video*-*activity score* assessed physical activity with r = 0.81 to the *movement rating* in the eyes of two independent observers. However, we were disillusioned by the missing relationship between the *video*-*activity score* and the *Hyperactivity* ratings from clinical experts. Moreover, we also observed no substantial association to the parental ratings of *Hyperactivity*. Finally, and unexpectedly, the age-related physical attributes showed a considerable dependence on our *video*-*activity score*. Most likely, we could only reduce, but not eliminate, the influence of age and physical attributes with our recording settings (see Fig. 1). These unexpected findings were again replicated by multiple regression, which balanced for all of the inequalities in all of the other included variables.

### Interpretation of the video-activity score

We interpret our findings that our *video*-*activity score* assesses physical activity (see *movement ratings*), which is mainly driven by age. Interestingly, the *movement ratings* show a trend towards association with the expert ratings (r = 0.31) of hyperactivity. It seems that age causes the majority of difference in physical activity [48], while hyperactivity represents a more specific and subtle interpretation of a human observer. This interpretation is in line with findings by Dane et al. [25], who found no significant differences in activity levels of children with ADHD (combined type and predominantly inattentive type) who were measured in their daily activity during a whole day of clinical assessment.

### Further research questions

Our *video*-*activity score* represents a first attempt to retain an activity score based on webcam footage. Currently, it is unclear if different objective activity scoring approaches will show convergent validity. We distinguished the assessment of physical activity from hyperactivity in this pilot-study. The proportion of both is unclear across all of the daily activities of a child. Most likely, this suggests that we should not ask about differences in activity quantity, but in differences of activity quality. Such questions can probably be answered by improved analytical software. This should be accompanied by the evaluation of the underlying reasons for a given specific behavior.

Outside of our context of hyperactivity, we see the potential of our approach to use the file size of a compressed video captured from a standardized setting to assess movement. Increased movements by patients in a psychotherapy setting may indicate the manifestation of important emotional processes. Capturing movement is also a necessity in sleep medicine. Our approach is able to assess movement without an attachment to the patient. A final advantage of our assessment method is that our *video*-*activity score* is readily available and can be conducted on existing video material post hoc.

### Limitations

The results are based on a typical, but small, sample size for a pilot study in this field. Furthermore, we did not validate our results with a matched control sample. Finally, we did not simulate a setting with other involved children (e.g., classroom situations).

## Conclusions

We provide a valid indicator for physical activity with our *video*-*activity score*. Yet, to date, we have failed to demonstrate criterion validity of hyperactivity within a standardized setting and a short observation time for hyperactivity-specific behaviors based on clinical expert ratings. Our method has nevertheless an essential advantage compared to other objective assessment methods. Our *video*-*activity score* permits validation by subjective ratings based on the same video footage. In the future, this advantage may afford a higher agreement with rating scales, which are also based on visual impressions of hyperactivity.

## Additional files

Additonal file 1:
**Table 1.** Samples test and measurement scores. **Table 2.** Test and measurement scores intercorrelation. **Table 3.** Multiple regression on video-activity score.
