# Supplementary material for: An objective measure of hyperactivity aspects with compressed webcam video
Source: Child Adolesc Psychiatry Ment Health. 2015 Sep 10;9:45. doi: 10.1186/s13034-015-0076-1 (PMC4565011; doi:10.1186/s13034-015-0076-1)
Supplement: Additonal file 1: — Table 1. Samples test and measurement scores. Table 2. Test and measurement scores intercorrelation. Table 3. Multiple regression on video-activity score. [file 13034_2015_76_MOESM1_ESM.docx]

## Table 1 – Description of the minimum (min), maximum (max), mean (M) and standard deviation (SD) of the activity score; movement ratings; and ADHD ratings (hyperactivity, inattention and impulsivity) by clinical experts and parents as well as children’s physical attributes for the Total, clinical ADHD and control group.

|  |  | **TOTAL** | | | | **ADHD** | | **CONTROL** | | **t-test^d^** | | | **Cohen’s** |
| --- | --- | --- | --- | --- | --- | --- | --- | --- | --- | --- | --- | --- | --- |
|  |  | **N=39** | | | | **N=21** | | **N=18** | |  | | |  |
| **SOURCE** |  | **Min** | **Max** | **MEAN** | **SD** | **MEAN** | **SD** | **MEAN** | **SD** | ***df*** | ***t*** | ***p*** |  |
| Webcam | Activity Score^c^ | 2,07 | 11,61 | 4,69 | 1,99 | 5,34 | 2,29 | 3,84 | 1,08 | 28,68 | -2,75 | ,010 | 0,59 |
|  | Movement Rating | 1,00 | 3,08 | 1,67 | 0,57 | 1,94 | 0,59 | 1,36 | 0,36 | 33,65 | -3,73 | ,001 | 0,84 |
| Clinical ratings^a^ | Inattention | 0,00 | 2,67 | 1,00 | 0,75 | 1,43 | 0,71 | 0,48 | 0,38 | 31,57 | -5,27 | ,000 | 1,18 |
|  | Hyperactivity | 0,00 | 2,80 | 0,75 | 0,81 | 1,14 | 0,85 | 0,27 | 0,41 | 30,10 | -4,14 | ,000 | 0,92 |
|  | Impulsivity | 0,00 | 3,00 | 0,86 | 0,92 | 1,26 | 0,95 | 0,35 | 0,57 | 33,32 | -3,65 | ,001 | 0,82 |
| Parental ratings^b^ | Inattention | 0,00 | 3,00 | 1,56 | 0,78 | 1,79 | 0,61 | 1,27 | 0,89 | 36,00 | -2,13 | ,040 | 0,48 |
|  | Hyperactivity | 0,00 | 2,86 | 1,11 | 0,8 | 1,22 | 0,81 | 0,98 | 0,78 | 36,00 | -0,90 | ,374 | 0,21 |
|  | Impulsivity | 0,00 | 3,00 | 1,34 | 0,93 | 1,51 | 0,97 | 1,12 | 0,86 | 36,00 | -1,31 | ,199 | 0,30 |
| Physical Attributes | Age | 6 | 16 | 11,08 | 3,17 | 9,19 | 2,89 | 13,28 | 1,74 | 33,47 | 5,43 | ,000 | 1,21 |
|  | Height | 120 | 180 | 149 | 17 | 140 | 14 | 160 | 14 | 37,00 | 4,60 | ,000 | 1,01 |
|  | Weight | 20,00 | 79,00 | 42,64 | 16,03 | 34,24 | 11,74 | 52,44 | 14,93 | 37,00 | 4,26 | ,000 | 0,96 |
|  | BMI | 11,48 | 31,22 | 18,59 | 4,36 | 17,08 | 3,24 | 20,36 | 4,91 | 37,00 | 2,50 | ,017 | 0,56 |

Note. ^a^DCL-ADHS. ^b^FBB-ADHS. ^c^128x96pixels, KB per second, **^d^** adjusted estimate in case of unequal variances.

Table 2 Intercorrelation between the video-based activity score, movement rating, expert (DCL-ADHS) and parental (FBB-ADHS) ratings for hyperactivity subscale and child age, weight, height and BMI (N=39).

| Source |  | Video | Expert | Parent | Physical Attributes | | | |
| --- | --- | --- | --- | --- | --- | --- | --- | --- |
|  |  | Movement Rating | Hyperactivity | Hyperactivity | Age | Height | Weight | BMI |
| Video | Activity score | ,81** | ,12 | ,20 | -,53** | -,43** | -,44** | -,37* |
|  | Movement Rating |  | ,31^t^ | .17 | -,58** | -.56** | -.57** | -.47** |
| Expert^1^ | Hyperactivity |  |  | ,48** | -,40* | -,40* | -,28^t^ | -,12 |
| Parent^1^ | Hyperactivity |  |  |  | -,32* | -,29^t^ | -,03 | ,18 |
| Physical  Attributes | Age |  |  |  |  | ,91** | ,80** | ,49** |
|  | Height |  |  |  |  |  | ,82** | ,42** |
|  | Weight |  |  |  |  |  |  | ,86** |
|  | BMI |  |  |  |  |  |  |  |

^t^ p < .10. *p < .05. **p < .01. ***p < .001

^1^ N=38

**Table 3** – Results of a multiple regression analysis to examine the influencing factors on the *video*-*activity score* based on the file size of compressed webcam footage of children in a standardized setting during a cognitive performance task (N=37).

|  |  | B | SE B | ß | t | p |
| --- | --- | --- | --- | --- | --- | --- |
| Video | Activity Score | 10,68 | 1,73 |  | 6,17 | ,000 |
| Physical | Age | -99,34 | 43,96 | -0,43 | -2,26 | ,031 |
| Attributes | BMI | -35,91 | 30,15 | -0,22 | -1,19 | ,242 |
| Expert Rating^a^ | Hyperactivity | 139,85 | 155,59 | -0,16 | -0,90 | ,375 |
| Parental Rating^b^ | Hyperactivity | 151,58 | 166,33 | 0,17 | 0,91 | ,369 |

Note. R^2^=.330 (p<.05). ^a^DCL-ADHS. ^b^FBB-ADHS.
